# Does variation of surgical technique affect native tissue anterior pelvic organ prolapse repair outcomes?

**DOI:** 10.1007/s00192-023-05584-4

**Published:** 2023-07-21

**Authors:** Emily Fairclough, Julia Segar, Suzanne Breeman, Anthony Smith, Jenny Myers, Fiona Reid

**Affiliations:** 1 Warrell Unit, St Mary’s Hospital, Manchester Foundation Trust, Oxford Road, Manchester, M13 9WL UK; 2 Maternal & Fetal Health Research Centre, Manchester Academic Health Centre, University of Manchester, Manchester, M13 9WL UK; 3 Institute of Population Health, University of Manchester, Oxford Road, Manchester, M13 9PL UK; 4 Health Services Research Unit, University of Aberdeen, 3rd Floor, Health Sciences Building, Foresterhill, Aberdeen, AB25 2ZD UK

**Keywords:** Pelvic organ prolapse, Native tissue, Technique, Outcome

## Abstract

**Introduction and hypothesis:**

The Variation in Surgical Technique study (VaST), demonstrated the large variation in surgical techniques used in native tissue (NT) anterior pelvic organ prolapse (POP) repairs. However, there are few comparative studies of different surgical techniques. This study was aimed at exploring whether surgical technique influenced the outcomes of NT anterior POP repairs.

**Methods:**

The surgical techniques of 22 consultant surgeons performing NT anterior POP repairs were filmed and categorised. These surgeons performed 809 anterior repairs within the PROlapse Surgery: Pragmatic Evaluation and randomised Controlled Trial (PROSPECT). Logistical regression models were used to determine the influence of the different surgical techniques on subjective and objective outcomes, using data collected during PROSPECT.

**Results:**

In adjusted multivariate linear regression models, fascial-flap repair was associated with an improved subjective outcome (POP-SS) compared with midline plication (β = −2.50 [−4.42 to −0.57]; *p* = 0.01). At 12 months, separate fascial defect repair was associated with a poorer objective outcome than midline plication (OR 6.06 [1.82–3.52], *p* = 0.006). At 24 months, deep dissection was associated with a poorer POP-SS than superficial dissection (0.32–2.60, *p* = 0.0). Continuous-locking closure of the skin was also associated with improved POP-SS compared with continuous non-locking closure (12 months: β = −1.94 [−3.42 to −0.45], *p* = 0.01).

**Conclusion:**

Surgical technique may influence the outcome of native tissue anterior POP repairs. Our results should not change practice but inform future research; to develop methods of explicitly recording surgical techniques and allow confirmation of the effect of these aspects of technique on outcome.

## Introduction

In 1909, White stated, “The only problem in plastic gynaecology left unresolved is the permanent cure of cystocele” [1]. Despite this being over 100 years ago, the recurrence of cystocele and its optimal management remain key concerns for surgeons today.

Failure rates for native tissue (NT) anterior pelvic organ prolapse (POP) repairs are reported to vary (37–97%) [2, 3]. This led to the use of polypropylene mesh to augment repairs. However, randomised controlled trials (RCTs) of mesh versus NT have found no benefit in the short term. Also, the complications of mesh [4] and the Cumberlege report [5] in the UK have led to a change in practice. Many surgeons no longer use mesh augmentation, preferring to perform NT repairs.

Several questionnaire studies have shown the variation in surgical techniques used in NT prolapse surgery [6–9]. Our study, Variation in Surgical Techniques (VaST), demonstrated that previous questionnaire studies may be flawed, as there are inconsistencies in the terminology used to describe surgical techniques (8). The effect of surgical technique variation on the outcome of surgery was not assessed in these studies. There are a lack of randomised trials comparing these different NT techniques [10]. Surgeons often “mix and match” different techniques making it difficult to categorise a specific NT operation.

In our previously published qualitative study, we described the variation in each step of the NT anterior POP repair procedure [11]. Hence, techniques were categorised based on each step of the procedure rather than the procedure as a whole. The aim of this study was to assess whether any step in the NT anterior POP repair procedure was associated with improved or worse subjective or objective outcomes, with a view to designing an RCT for NT repair techniques.

## Materials and methods

Ethical approval was gained from the Sunderland Ethics Committee (REC number: 13/NE/0158).

A qualitative study (VaST) [11] used video-taped observations of surgery and audio-taped interviews with surgeons to evaluate the surgical techniques used to perform NT anterior POP repairs by a cohort of 30 consultant surgeons. The study group was a purposive sample drawn from a cohort of consultant surgeons who had recruited to a large surgical prolapse study, the PROlapse Surgery: Pragmatic Evaluation and randomised Controlled Trial (PROSPECT) [4] and a sample of non-PROSPECT consultant surgeons, to ensure a representative sample. Recruitment concluded following saturation of the themes. Thematic analysis was performed [12] and Table 1 outlines the themes of surgical technique identified.

**Table 1:** Themes of surgical technique and the frequency/percentage of anterior native tissue repairs in each theme

Theme	Sub themes	Number of women	Percentage
Depth of fascial dissection	Superficial	482/724	67
	Deep	242/724	33
Method of fascial repair	Midline	611/809	75
	Ultra-lateral	61/809	8
	Separate fascial defect	50/809	6
	Skin, i.e. no fascial repair	37/809	5
	Fascial flap repair	50/809	6
Fascial suture placement	Midline central	157/722	22
	Midline lateral	504/722	70
	Ultra-lateral	61/722	8
Number of fascial layers	1	588/759	77
	≥ 2	171/759	23
Fascial suture material	PGA	411/809	51
	PDS	347/809	43
	PGA + PDS	51/809	6
Fascial suture method	Continuous	293**/**759	39
	Continuous locking	78/759	10
	Interrupted	388**/**759	51
Skin trimming	Yes	139/809	17
	No	670/809	83
Skin suture material	PGA	751/809	93
	Monocryl	58/809	7
Skin suture method	Continuous	84/809	10
	Continuous locking	725/809	90

*PGA* polyglycolic acid, *PDS* polydioxanone suture

During the PROSPECT study, surgeons were requested to use the surgical technique they used most often in clinical practice. The current study therefore performed a nested analysis, which was aimed at quantifying the association between the different surgical technique themes, generated in the qualitative study (VaST), and patient outcomes collected as part of PROSPECT. PROSPECT outcomes were included for women who had a primary NT anterior POP repair performed by one of the subgroup of consultant surgeons (*n* = 22) who participated in the VaST study.

Data from women operated on by two of the VaST study surgeons were excluded as they had only performed one procedure each within the PROSPECT trial. In the theme “depth of dissection”, data relating to two surgeons (85 operations) were excluded, as the technique could not be categorised as deep or superficial. Women operated on by surgeons who routinely placed sutures in the skin rather than in the fascia (*n* = 37) and those who performed separate fascial defect repairs (*n* = 50), had data excluded from the themes fascial suture placement and number of fascial layers as the sutures were not placed in the fascia or the placement/layers varied depending on the defect.

The investigator (JM) performing the statistical analysis was blinded to the identity of the surgeons in each theme. The subjective outcome measure used in this study was the Pelvic Organ Prolapse Symptom Score (POP-SS) [13]. Both 12- and 24-month data were analysed and the distribution of scores normalised by the removal of 3 outliers with a score > 10. The objective outcome measure, assessed at 12 months after surgery, was the POP-Q measurement Ba. The postoperative Ba measurement was dichotomised into cure (above the hymen) and failure (beyond the hymen).

The data were checked for normality and transformed where necessary prior to analysis. For each outcome measure, a potential association with all relevant patient/procedure characteristics (age, parity, body mass index [BMI], primary or secondary repair, inclusion in the RCT or cohort study, concomitant surgery (posterior repair and vault or incontinence procedure)) was assessed using linear or logistic regression models as appropriate. Characteristics with borderline or significant association (*p* ≤ 0.1) with the outcome variable were included in the models investigating the effect of the themed analysis for that outcome. The association between each of the surgical themes and the subjective and objective outcomes was then investigated using mixed effects regression models (to account for surgeon clusters).

The potential clustering effect of the individual surgeon was quantified using mixed effects regression; an intraclass coefficient (ICC) of > 10% was considered a significant effect of individual surgeon. Owing to sample size limitations the effect of each theme was assessed individually; the effect associated with individual surgeon was therefore used as a proxy to adjust for the simultaneous variation in the other themes. A POP-SS change of −2 or more was considered clinically significant [13]. Significance was set at *p* ≤ 0.01 to account for multiple testing. Owing to the novel and exploratory nature of the analysis, an a priori sample size calculation was not possible.

## Results

The 20 consultant surgeons included in this study performed a total of 809 NT anterior POP repairs within PROPSECT. These procedures were performed alone (*n* = 186) or in combination with concomitant procedures including posterior repairs, vault and continence (*n* = 623). The demographics of these women are shown in Table 2.

**Table 2:** Demographics of women

Covariant	Median	Interquartile range (p25–p75)
Age	51	53–68
Body mass index	27	24–31
Parity	2	2–3

### Subjective outcome score: POP-SS

Overall, at 12 and 24 months, the median change in POP-SS score was −8 (range −12 to −4). A clinically significant change (improvement) in outcome is demonstrated by a reduction in score by 2 points [13]. These data demonstrate that the majority of women reported subjective improvement following surgery.

#### Subjective outcome: confounding variables

The patient/procedure characteristics that were identified as having some association with the subjective outcome were concomitant posterior repair (improved POP-SS) (12 months: β −0.79 [95% CI −1.85 to 0.27], *p* = 0.146; and 24 months: β −0.98 [95% CI −2.09 to 0.14], *p* = 0.088) and incontinence procedure (poorer POP-SS; 12 months: β 1.67 [95% CI −0.23 to 3.50], *p* = 0.085; and 24 months: β 1.86 [95% CI −0.15 to 3.8], *p* = 0.07). The impact of individual surgeons was negligible at both time points (ICC 0.02 [95% CI 0.006 to 0.09], ICC 0.03 [95% CI 0.01–0.11]). The other confounding variables, (surgeon, age, parity, BMI, primary or secondary repair, cohort or RCT and concomitant vault prolapse) had no significant impact on subjective outcome.

#### Subjective outcome: 12-month time point

At 12 months, the majority of themes of technique routinely used by surgeons in the steps of the anterior repair procedure did not significantly affect the subjective outcome (POP-SS; Table 3). Women who were operated on by a surgeon who routinely employed a fascial-flap repair (*n* = 43) compared with a midline repair of the vaginal muscularis (fascia; *n* = 477) had a significantly improved POP-SS (β −2.49 [95% CI −4.42 to −0.57], *p* = 0.01). This did not change significantly following adjustment for surgeon (β −2.66 [95% CI −4.89 to −0.44], *p* = 0.019; ICC 0.006 [0.0001–0.24]); however, the fascial flap technique was only performed by one surgeon. In women whose surgeon routinely used a continuous locking method of skin closure (*n* = 579) compared with continuous non-locking skin closure (*n* = 77) there was also a significant improvement in POP-SS (β −1.94 [95% CI −3.43 to −0.45], *p* = 0.01; Table 3).

**Table 3:** The effect of surgical technique on subjective and objective outcomes

		Symptomatic outcome: POP-SS						Objective outcome: Ba		
		12-month data			24-month data			12-month data		
Category (comparative theme)	Theme (number of surgeons)	*p* value	95% CI	Coefficient	*p* value	95% CI	Coefficient	*p* value	95% CI	OR
**Significant findings**										
Depth of fascial dissection (superficial *n* = 12)	Deep (*n* = 6)	0.66	−1.36–0.86	−0.24	**0.01**	0.32–2.60	1.47	0.44		0.44
Fascial repair method (midline *n* = 14)	Ultra-lateral (*n* = 2)	0.55	0.55	−0.52	0.055	−3.48–0.03	−1.72	0.69	0.30	1.37
	Separate fascial defect (*n* = 1)	0.70	0.70	0.36	0.068	−3.59–0.12	−1.73	**0.006**	1.82–35.26	6.06
	Skin (*n* = 2)	0.25	0.25	1.36	0.170	−3.99–0.70	−1.64	Number too low	1.82	Number too low
	Fascial dlap (*n* = 1)	**0.01**	−4.42–−0.57	−2.49	**0.001**	−5.33–1.34	−3.34	0.95	0.18	1.02
Skin suture method (continuous non-locking *n* = 2)	Continuous locking (*n* = 18)	**0.01**	−3.43–−0.45	−1.94	0.02	−3.43–0.37	−1.91	0.77	0.13–4.43	0.77
**Non-significant findings**										
Fascial suture placement (midline central *n* = 2)	Midline lateral (*n* = 13)	0.96	1.3	−0.31	0.15	2.4	1.05	0.78	0.21–3.26	0.82
	Ultra-lateral (*n* = 2)	0.71	1.0	−0.38	0.69	1.7	−0.44	0.88	0.15–8.82	1.17
Number of fascial layers (1, *n* = 15)	2 + (*n* = 4)	0.37	1.7	0.54	0.82	1.3	0.14	0.25	0.16–1.6	0.25
Fascial suture material (PGA, *n* = 8)	PDS (*n* = 10)	0.26	1.5	0.57	0.20	1.7	0.68	0.05	0.12–0.99	0.35
	PDS + PGA (*n* = 2)	0.15	3.4	1.45	0.091	−0.30–4.0	1.86	0.141	0.12–0.98	0.27
Fascial suture method (continuous non-locking *n* = 6)	Continuous locking (*n* = 1)	0.86	1.9	0.16	0.351	0.96	−0.87	0.400	0.34–14.1	2.21
	Interrupted (*n* = 12)	0.08	−1.96–9.1	−0.93	0.11	0.19	−0.91	0.27	0.61–5.74	1.87
Skin trimming (No, *n* = 3))	Yes (*n* = 17)	0.87	1.40	0.11	0.06	−0.04–2.6	1.31	0.53	0.13–2.84	0.60
Skin suture material (PGA, *n* = 19)	Monocryl (*n* = 1)	0.14	3.1	1.35	0.05	−0.02–3.7	1.86	0.72	0.14–1.24	1.57
Pack (no, *n* = 6)	Yes (*n* = 15)	0.4			0.74			0.99		

**Bold outcomes** = significant findings

**Fig. 1:**
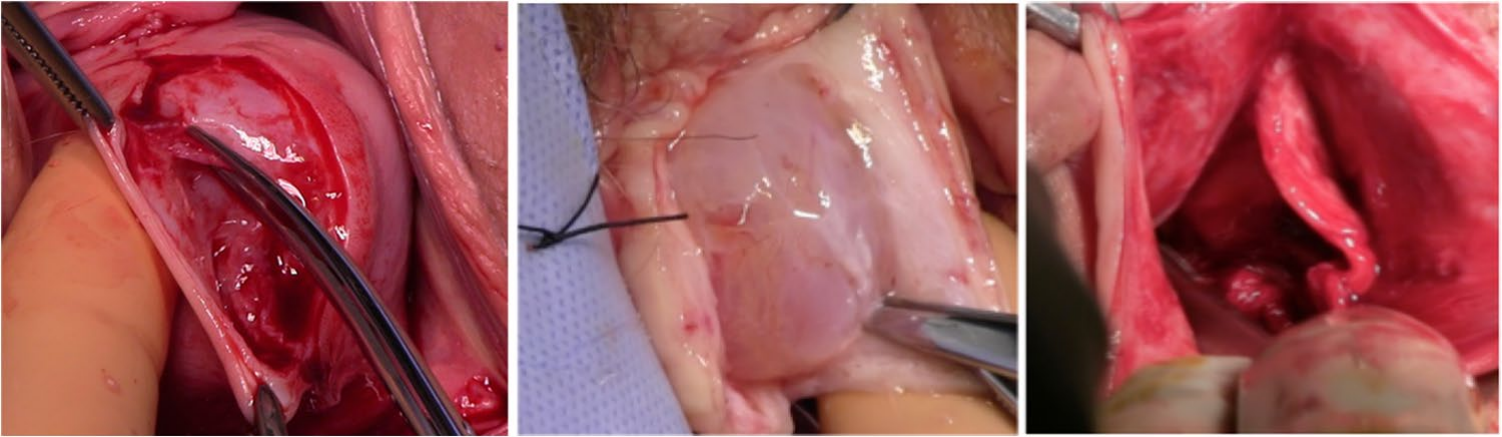
Photographic illustrations of the methods of fascial dissection. **a** Superficial dissection, **b** deep dissection, **c** fascial-flap dissection

Figure 1c illustrates dissection of the vaginal muscularis from the vaginal epithelium and the underlying bladder, creating “fascial flaps”. Sutures were placed in the vaginal muscularis and it was brought together in the midline. For a full explanation of the different techniques performed in the qualitative study, see the earlier paper in this series [11].

#### Subjective outcome: 24-month time point

At 24 months, again, the majority of surgical technique themes did not significantly affect the subjective outcome (POP-SS). Consistent with the 12-month outcome data, a fascial-flap repair method (*n* = 37) appeared to be associated with improved POP-SS compared with midline repair (*n* = 404; β −3.34 [95% CI −5.33 to −1.34], *p* = 0.001). The continuous-locking method of skin closure was also associated with improved POP-SS compared with the continuous non-locking method (β −1.91 [−3.43 to −0.37], *p* = 0.01); however following adjustment for surgeon, this did not reach clinical or statistical significance (*p* = 0.05).

In women whose surgeon routinely used the deep-dissection technique (below the vaginal muscularis; Fig. 1b; *n* = 242) compared with superficial dissection (between the vaginal epithelium and vaginal muscularis; Fig. 1a; *n* = 482), there was a worsening of the POP-SS (β 1.47 [95% CI 0.32 to 2.60], *p* = 0.01). This effect was not changed following adjustment for individual surgeon.

### Objective outcome: the most dependent part of the anterior wall (Ba)

The most dependent part of the anterior compartment prolapse (Ba) was measured postoperatively at 12 months in a subgroup of women who participated in the randomised component of PROSPECT (*n* = 276). Those in the comprehensive cohort study completed questionnaire data only. The number of patients having an objective failure (Ba at or below the level of the hymen) was 83 (30%).

#### Objective outcome: confounding variables

Concomitant posterior repair was associated with a reduction in objective failure (OR 0.54 [95% CI 0.30–0.96], *p* = 0.04); in addition, there was a significant effect of surgeon (ICC 0.16 [95% CI 0.050 to 0.42]). Both characteristics were therefore included in the subsequent analysis. The other confounding variables (age, parity, BMI, primary or secondary repair, cohort or RCT and concomitant vault support/incontinence procedure) had no significant impact on the objective outcome.

#### Objective outcome: 12-month time point

Most themes of technique were not significantly associated with objective failure at 12 months (Table 3). The likelihood of objective failure was higher in women who were operated on by a surgeon who routinely performed separate fascial defect repairs compared with midline repair of the vaginal muscularis (fascia; OR 6.06 [95% CI 1.82 to 35.26], *p* = 0.006). When surgeons routinely used polydioxanone suture (PDS) to repair the vaginal muscularis this was associated with proportionally more women being cured (75 out of 92) compared with polyglycolic acid (PGA) suture (95 out of 156; OR 0.35 [95% CI 0.12–0.99], *p* = 0.05), but this finding was not statistically significant (Table 3).

### Complications

The frequency of complications (wound infection and haematoma) was small and therefore formal statistical comparison was not possible. Six percent of women (49 out of 809) had a wound infection postoperatively. The numbers of women who had a wound infection in the RCT and cohort were 28 out of 312 and 21 out of 448 respectively. BMI and being part of the cohort group (compared with the RCT) were weakly associated with this complication. There was no effect of the surgeon (ICC < 0.01) on the rate of infection. No aspect of surgical technique was associated with the wound infection rate. Eight patients (1%) had a haematoma postoperatively. In all 8 cases, continuous-locking skin sutures made of PGA were used. Considering these 8 patients, 4 (50%) had had a pack inserted following the operation and 4 (50%) had not.

## Discussion

The VaST study found aspects of surgical technique that may influence outcome and warrant further research. This was an exploratory study assessing the influence of technique in each step of the NT anterior repair procedure. The majority of themes of technique did not influence outcome; however, the results present aspects of the operation that could influence outcome and should be further explored.

The themes of technique that were shown to influence outcome included the depth of dissection, method of fascial (vaginal muscularis) repair and skin-suturing technique. Women whose surgeon routinely used the deep-dissection technique had a worse subjective outcome than those who used superficial dissection, but this was not supported by a difference in the objective outcome. The fascial-flap repair, which was used by a single surgeon, was associated with an improved subjective outcome, but again, not an improved objective outcome. Continuous-locking suturing of the skin showed an improved subjective outcome compared with continuous non-locking at 12 months but not at 24 months. No aspect of the technique was found to have a positive effect on objective outcome. A separate fascial defect repair was associated with a poorer objective outcome, but the effect on subjective outcome was not significant.

Even using the large set of NT anterior POP repair outcome data from PROSPECT, the extent of variation in surgical technique found amongst surgeons means that the results of VaST must be interpreted with caution. Furthermore, some techniques were only performed by one surgeon, so there will be other confounding factors influencing outcomes. The stage of prolapse prior to surgery was not accounted for and could have introduced some bias. Nevertheless, VaST remains an important study for directing further research to determine the best method of NT anterior POP repair. The study highlights the need to standardise and make explicit the descriptions of the surgical techniques used during prolapse surgery. This is important because repeated studies have demonstrated that the current techniques employed do not result in a satisfactory outcome for a third of women.

To our knowledge, this is the first study of variation in surgical technique, in NT anterior POP repair, to combine the assessment of technique at each step of the procedure with the outcome of surgery. Steps were taken to ensure the rigour of the qualitative study [11]; these included triangulation of methods (video-recorded observations, audio-recorded interviews and field notes), immediate validation of the observed data through subsequent surgeon interviews, recruitment completion beyond saturation point and the involvement of multiple investigators to review and categorise data. An independent investigator who was blinded to the themes of surgical technique performed the statistical analysis of the outcome data from PROSPECT.

All PROSPECT surgeries were carried out before the surgeons were interviewed. PROSPECT was a pragmatic trial; hence, surgeons were requested to use their routine surgical technique for native tissue anterior POP repair throughout the trial [8]. However, aspects of technique could have varied between patients, which could have introduced some bias.

As this was an exploratory study, calculation of a formal sample size was not possible. The outcome data used were powered to assess the influence of native tissue and mesh repairs on the subjective outcome (POP-SS). As this was a subgroup analysis, the assessment of some of the surgical themes will inevitably be underpowered and a significant effect on outcome could have been missed. The majority of themes were performed in at least 10% of the cases investigated; however, in some instances by only one surgeon. We attempted to account for the potential confounding effect attributable to different combinations of surgical themes and/or experience of the surgeon by quantifying the clustering (ICC) effect of individual surgeons. In the majority of the analyses the ICC was low, which would support only a small contribution to the association with outcome by an individual surgeon. However, it is possible that sample size limitations have resulted in a significant clustering effect being missed.

Although the results must be interpreted with caution owing to the large number of variables, the study found that subjective outcomes were better following fascial flap techniques and worse following deep dissection; however, this was not reflected in the objective outcomes. Others have reported that objective recurrence of prolapse does not always equate with the return of symptoms [14, 15]. It could be hypothesised that techniques that result in greater tissue disruption, such as developing fascial flaps, cause increased denervation and this results in a reduction of awareness of bulge, whereas a deep dissection performed in an avascular plane may be nerve sparing and may therefore result in greater subjective awareness of failure.

To our knowledge, this is the first study to relate the techniques in every step of an anterior POP repair procedure with outcome. Previous studies have looked at the effect of single aspects of technique [16–20]. One previous randomised control trial of native tissue fascial repair techniques compared two techniques, “standard colporrhaphy” and “ultra-lateral colporrhaphy”. They also found similar objective cure rates [10] and symptom resolution [16] for both techniques. It may be that patient factors, not yet identified, have a significant impact on the outcome of surgery. However, they did not assess the effect of other techniques of fascial repair, fascial-flap repair and separate fascial defect repairs, identified in this study. (Of note, some readers may prefer the term vaginal muscularis to fascia but there is no internationally recognised agreement about the use of either term).

The “deep-dissection technique”, in which the incision is performed at the level of the vaginal adventitia, is likely to have been extrapolated by surgeons from techniques they learnt when inserting mesh [21]. Its use for native tissue repair has not been previously published and yet several surgeons in our cohort have adopted it in their routine clinical practice. This demonstrates the way in which surgeons evolve their techniques in an experiential manner, rather than through evidence-based practice.

This article highlights one of the challenges of studying prolapse surgery outcomes: there is no “standard” NT anterior POP repair. Our qualitative study [11] clearly outlines all variations in surgical technique, at all steps of the procedure and demonstrates that there remains tacit aspects of surgery that are still difficult to quantify and record. Whether surgical techniques can be explicitly described and standardised remains uncertain. Hence, large pragmatic surgical trials such as PROSPECT are important because their results reflect surgical techniques in the “real world”, with all their inherent variables.

In conclusion, surgical technique may influence the outcome of NT anterior POP repairs. These results should not change current individual surgeons’ techniques but inform future research, developing methods of explicitly recording techniques and designing studies to confirm whether different aspects of technique influence the subjective and objective outcomes of NT anterior POP repairs.

## Data Availability

Data can be made available upon reasonable request to author JM and the University of Manchester.

## Abbreviations

ICC: Intraclass coefficient
NT: Native tissue
POP: Pelvic organ prolapse
POP-SS: Pelvic organ prolapse symptom score
RCT: Randomised controlled trial
